# N-Cadherin Negatively Regulates Osteoblast Proliferation and Survival by Antagonizing Wnt, ERK and PI3K/Akt Signalling

**DOI:** 10.1371/journal.pone.0008284

**Published:** 2009-12-14

**Authors:** Eric Haÿ, Alexandra Nouraud, Pierre J. Marie

**Affiliations:** Laboratory of Osteoblast Biology and Pathology, INSERM UMR 606 and University Paris Diderot, Paris, France; University of Texas MD Anderson Cancer Center, United States of America

## Abstract

**Background:**

Osteoblasts are bone forming cells that play an essential role in osteogenesis. The elucidation of the mechanisms that control osteoblast number is of major interest for the treatment of skeletal disorders characterized by abnormal bone formation. Canonical Wnt signalling plays an important role in the control of osteoblast proliferation, differentiation and survival. Recent studies indicate that the cell-cell adhesion molecule N-cadherin interacts with the Wnt co-receptors LRP5/6 to regulate osteoblast differentiation and bone accrual. The role of N-cadherin in the control of osteoblast proliferation and survival remains unknown.

**Methods and Principal Findings:**

Using murine MC3T3-E1 osteoblastic cells and N-cadherin transgenic mice, we demonstrate that N-cadherin overexpression inhibits cell proliferation *in vitro* and *in vivo*. The negative effect of N-cadherin on cell proliferation results from decreased Wnt, ERK and PI3K/Akt signalling and is restored by N-cadherin neutralizing antibody that antagonizes N-cadherin-LRP5 interaction. Inhibition of Wnt signalling using DKK1 or Sfrp1 abolishes the ability of N-cadherin blockade to restore ERK and PI3K signalling and cell proliferation, indicating that the altered cell growth in N-cadherin overexpressing cells is in part secondary to alterations in Wnt signalling. Consistently, we found that N-cadherin overexpression inhibits the expression of Wnt3a ligand and its downstream targets c-myc and cyclin D1, an effect that is partially reversed by N-cadherin blockade. We also show that N-cadherin overexpression decreases osteoblast survival *in vitro* and *in vivo*. This negative effect on cell survival results from inhibition of PI3K/Akt signalling and increased Bax/Bcl-2, a mechanism that is rescued by Wnt3a.

**Conclusion:**

The data show that N-cadherin negatively controls osteoblast proliferation and survival via inhibition of autocrine/paracrine Wnt3a ligand expression and attenuation of Wnt, ERK and PI3K/Akt signalling, which provides novel mechanisms by which N-cadherin regulates osteoblast number.

## Introduction

Wnt proteins are a family of secreted proteins that play important roles in the development and maintenance of many tissues [1]. Wnt proteins control cell proliferation, differentiation and survival through signals involving β-catenin-dependent and -independent pathways [2], [3], [4], [5]. Binding of canonical Wnts to the 7-transmembrane domain-spanned frizzled (Fz) receptor and low-density lipoprotein 5 and 6 (LRP5/6) co-receptors initiates a cascade of events triggered by the cytoplasmic protein Dishevelled (Dsh) interacting with Fz, Axin and Frat-1. Disruption of this complex leads to phosphorylation of GSK-3β and inhibition of β-catenin phosphorylation. This effect results in β-catenin stabilization and its subsequent translocation into the nucleus where it interacts with TCF/LEF transcription factors to activate the expression of Wnt-responsive genes [6]. Wnt signalling is tightly regulated by secreted regulatory proteins. Soluble frizzled-related proteins (Sfrps) and WIF-1 antagonize Wnt-Fz interactions whereas Dickkopf (Dkk) antagonizes LRP5/6 [7]. Wnt signalling is also controlled by intracellular antagonists such as Axin, APC and Groucho which regulate β-catenin stability and activity [6], allowing fine control of signals triggered by Wnt proteins [8]. Wnt proteins also control kinase signalling pathways. Notably, canonical Wnt3a increases PI3K/Akt activity, resulting in GSK3β phosphorylation and increased free β-catenin levels [9]. In addition, Wnt3a activates ERK1/2 by direct signalling and posttranscriptional activation via the β-catenin/Tcf4 complex [10], indicating that these kinases may act as important mediators of Wnt signalling.

In the recent years, canonical Wnt signalling has emerged as an important regulator of bone formation and bone mass [11], [12], [13], [14], [15]. The importance of Wnt signalling in the control of bone mass was initially demonstrated by the high and low bone mass phenotype caused by loss- and gain-of-function LRP5 mutations, respectively [16]. Further evidence for the important role of Wnt signalling in bone was provided by the changes in bone mass caused by inactivation or overexpression of Wnt antagonists in the mouse [17]. Recent data indicate that the skeletal effects of LRP5 may be indirect and mediated by gut-derived serotonin [18]. This does not rule out however that LRP5/6 may have direct skeletal effects at early stages of the osteoblast lineage [19]. *In vitro*, Wnt signalling positively controls osteoblast differentiation by activating the Wnt/LRP5/β-catenin/LEF-TCF/Runx2 signalling cascade [20]. In addition, Wnt signalling controls cell proliferation during progression along the osteogenic lineage. Stable expression of Wnt proteins or LRP5 enhances osteoblast progenitor cell growth *in vitro* [21], [22]. Consistently, LRP5 deficiency results in reduced osteoblast proliferation in mice [23]. Furthermore, Wnt signalling was found to prevent apoptosis in uncommitted osteoblast progenitors and more mature osteoblasts [24]. Accordingly, a gain-of-function mutation in LRP5 (G171V) decreases osteoblast/osteocyte apoptosis [25] whereas deletion of the Wnt antagonist Sfrp1 reduces osteoblast apoptosis [12]. These effects are mediated in part via the Wnt/β-catenin canonical pathway [26], [27], [28]. However, prevention of apoptosis in uncommitted osteoblasts and mature osteoblasts by Wnt proteins may also occur through activation of Src/ERK and PI3K/Akt pathways [24], indicating that multiple pathways are involved in the control of osteoblast proliferation and survival by Wnt proteins.

Cadherins are cell-cell adhesion molecules that mediate cellular signalling [29], [30], [31]. Previous studies indicate that cadherins interact with Wnt signalling by sequestering β-catenin at the plasma membrane [29], [32], [33]. In bone, N-cadherin is strongly expressed in osteoblasts and regulates osteoblast differentiation [34], [35] and bone mass [36], [37], [38] although the underlying mechanisms are not fully understood. We recently showed that N-cadherin interacts with LRP5/6 and negatively regulates Wnt signalling through β-catenin degradation, resulting in decreased osteoblast differentiation and bone formation *in vivo* [39]. However, the role of N-cadherin in the control of osteoblast proliferation and survival remains unknown.

Here we investigated the molecular mechanisms involved in the control of osteoblast growth and apoptosis by N-cadherin. We provide here novel evidence that N-cadherin acts as a negative regulator of cell proliferation and survival in osteoblasts via interaction with LRP5, alteration of autocrine Wnt3a ligand expression and attenuation of Wnt, ERK and PI3K/Akt signalling pathways.

## Results

**Figure 1:**
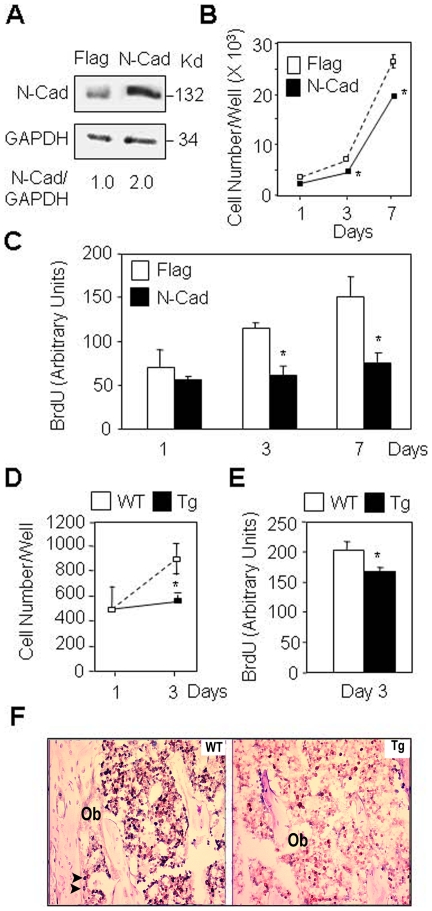
Enforced expression of N-cadherin decreases cell proliferation in osteoblasts. (A) MC3T3-E1 osteoblasts stably transfected with N-cadherin (N-Cad) display a 2-fold increase in N-cadherin expression compared to control cells (Flag) as shown by western blot analysis. (B, C) Decreased cell number and replication in N-Cad cells compared to Flag cells. (D, E) Decreased cell number and replication in primary calvarial osteoblasts isolated from N-cadherin transgenic mice (Tg) compared to osteoblasts from wild-type mice (WT). Means are +/− SD. Values that are significantly different are indicated (*, *P*<0.05 vs Flag or WT cells). (F) Histologial sections of tibias showing decreased cell proliferation in N-Cad Tg mice compared to WT mice, as revealed by Ki67 staining (black nuclei) in bone marrow stromal cells and mature osteoblasts (Ob, arrows) (x250).

The efficiency of N-cadherin overexpression in MC3T3-E1 osteoblastic cells was first checked by western blot analysis. A 2-fold increase in N-cadherin protein level was documented in N-cadherin-transfected MC3T3-E1 cells compared to control (Flag) cells (Figure 1A). We then determined the effect of N-cadherin overexpression on cell proliferation. As shown in Figure 1B, cell number was lower in N-cadherin overexpressing cells compared to control cells. This effect was in part related to a 50% decrease in cell replication, as shown by the BrdU assay (Figure 1C). To determine whether this negative effect of N-cadherin overexpression may be relevant *in vivo*, primary calvaria osteoblasts were isolated from 1.5 month old N-cadherin transgenic and wild-type mice and cell growth was assessed *ex vivo* by cell number and BrdU assay. As shown in Figure 1D, cell number was reduced in N-cadherin transgenic osteoblasts compared to wild-type osteoblasts. This effect was in part related to a lower cell replication in transgenic osteoblasts (Figure 1E), suggesting a cell autonomous defect in cell proliferation. We then performed an *in vivo* analysis of cell proliferation in bones from 1.5 month old N-cadherin transgenic mice. Cell proliferation detected by Ki67 staining in the bone marrow stroma (black nuclei) and in osteoblasts (arrows) was decreased in tibias of N-cadherin transgenic mice compared to wild type mice (Figure 1F). The decrease in cell proliferation observed in the bone marrow stroma of N-cadherin transgenic mice may be the consequence of alteration of endogenous Wnt3a expression (see below). These results show that increasing N-cadherin expression in osteoblasts results in decreased cell proliferation *in vitro* and *in vivo*.

**Figure 2:**
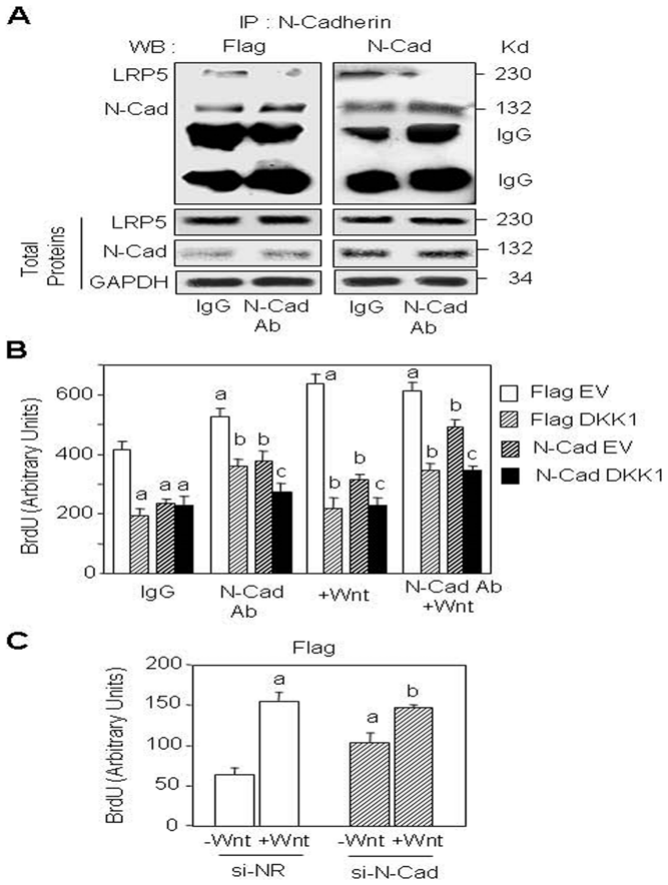
N-cadherin reduces cell proliferation via interaction with LRP5 and Wnt signalling. (A) Immunoprecipitation analysis showing N-cadherin and LRP5 interaction in control (Flag) cells and N-cadherin (N-Cad) overexpressing cells which is blocked by N-cadherin antibody. Cells were treated with blocking N-cadherin antibody (Ab) or control antibody (IgG) for 24 hours, cell lysates were immunoprecipitated (IP) with N-cadherin antibody and analysed by Western-blot (WB) with LRP5 antibody. LRP5, N-cadherin and GAPDH in total proteins were used as loading controls. (B) Treatment with Wnt3a or blocking N-cadherin antibody restores cell proliferation in N-Cad cells. Flag and N-Cad cells were transfected with DKK1 expression vector or empty vector (EV), treated with Wnt3a CM (15%) or N-cadherin antibody for 24 hours and cell replication was determined. Means are +/− SD. Values that are significantly different are indicated (a, *P*<0.05 vs untreated Flag EV cells; b, *P*<0.05 vs Flag EV cells treated with N-Cad Ab or Wnt3a; c, *P*<0.05 vs N-Cad EV cells treated with N-Cad Ab or Wnt3a). (C) N-cadherin silencing increases osteoblast proliferation. Flag cells were transfected with a specific N-cadherin si-RNA or a non relevant si-RNA (si-NR) and treated with Wnt3a CM (15%) for 24 hours and cell replication was determined (a, *P*<0.05 vs -Wnt si-NR treated cells; b, *P*<0.05 vs -Wnt si-N-Cad treated cells).

One mechanism by which N-cadherin may affect cell proliferation is by interacting with LRP5/6 [39]. Using immunoprecipitation and Western blot analyses, we confirmed that LRP5 interacts with N-cadherin in normal (Flag) osteoblasts and that this interaction is increased in N-cadherin overexpressing cells (Figure 2A). Neutralization of N-cadherin using a specific N-cadherin antibody that recognizes the extracellular domain of N-cadherin [40] efficiently decreased LRP5 level associated with N-cadherin (Figure 2A). We therefore used this tool to analyse the role of N-cadherin-LRP5 interaction on cell proliferation. As shown in Figure 2B, Wnt3a (15% CM) increased cell proliferation in both Flag and N-cadherin overexpressing cells and this effect was suppressed by DKK1, a high affinity ligand for LRP5/6 [41] that inhibits canonical Wnt signalling [42]. The neutralizing N-cadherin antibody also increased cell replication and the response to Wnt and these effects were blocked by DKK1 (Figure 2B). These results indicate that the decreased cell proliferation induced by forced expression of N-cadherin results from increased N-cadherin-LRP5 interaction and subsequent alteration of Wnt signalling. To investigate whether endogenous N-cadherin affects cell proliferation in osteoblasts, we used a specific N-cadherin si-RNA that efficiently reduces N-cadherin expression [39]. N-cadherin silencing using this si-RNA increased cell proliferation in control (Flag) cells compared to a non-relevant siRNA in the presence or absence of Wnt (Figure 2C). These results show that endogenous N-cadherin as well as forced expression of N-cadherin negatively regulates cell proliferation in osteoblasts.

**Figure 3:**
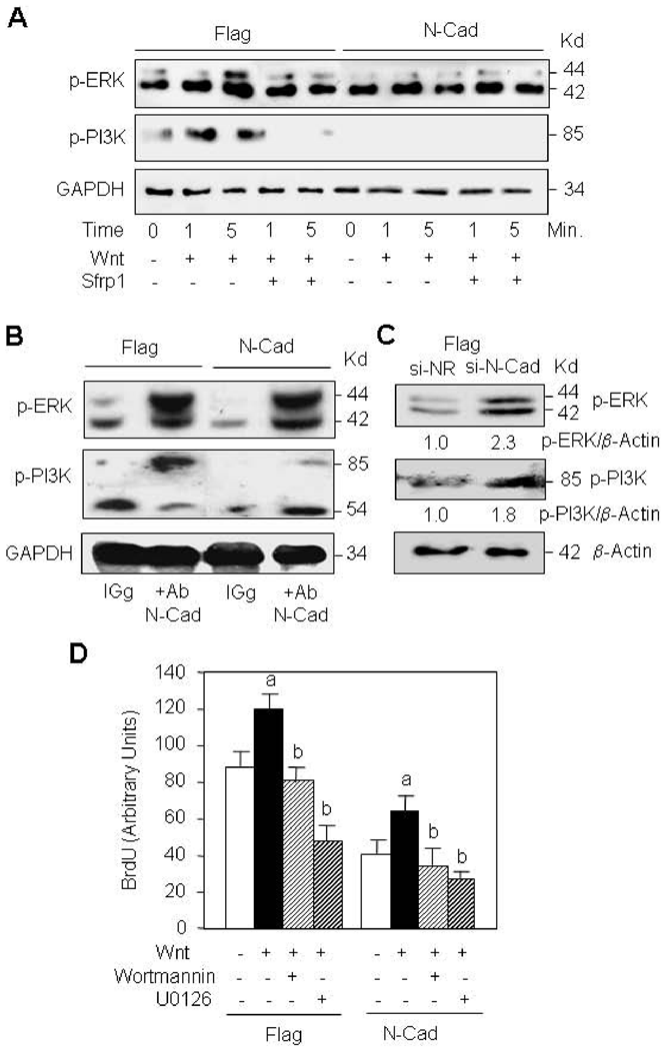
N-cadherin overexpression negatively regulates ERK and PI3K signalling. (A) Control (Flag) and N-cadherin (N-Cad) overexpressing cells were treated with canonical Wnt3a CM (15%) for 1 or 5 minutes and ERK and PI3K signalling was analysed by Western-blot. GAPDH was used as loading control. (B) N-cadherin blockade restores cell signalling in N-cadherin overexpressing osteoblasts. Flag and N-Cad cells were treated with N-cadherin antibody or control antibody (IgG) for 5 min and ERK and PI3K signalling was analysed by Western-blot. GAPDH was used as loading control. (C) N-cadherin silencing increases ERK and PI3K signalling. Flag cells were transfected with a specific N-cadherin si-RNA or a non relevant si-RNA (si-NR) and phospho-ERk and phospho-PI3K levels determined by western blot analysis were quantified using β-actin as loading control. (D) Treatment with PI3K and MEK inhibitors (Wortmannin and U0126, respectively) abolished cell proliferation induced by Wnt3a CM (15%) in both Flag and N-Cad cells at 24 hours. Means are +/− SD. Values that are significantly different are indicated (a, *P*<0.05 vs untreated cells; b, *P*<0.05 vs Wnt3a-treated cells).

We then investigated the signalling pathways underlying the inhibition of cell proliferation induced by N-cadherin. We focused on ERK and PI3K that are most important signalling pathways involved in cell growth. As shown in Figure 3A, western blot analysis showed that p-PI3K and p-ERK (p44) levels were decreased in N-cadherin overexpressing cells compared to control (Flag) cells in basal conditions. Wnt3a (15% CM) increased PI3K and ERK (p44) phosphorylation in Flag cells, an effect that was prevented by the Wnt inhibitor Sfrp1. In contrast, Wnt had no effect on ERK and PI3K phosphorylation in N-cadherin overexpressing cells (Figure 3A), indicating that PI3K and ERK signalling is altered in basal condition and in response to Wnt3a. To confirm this finding, cells were treated with the N-cadherin neutralizing antibody. The N-cadherin antibody increased p-ERK (mainly p44) and p-PI3K levels in both control (Flag) cells and N-cadherin overexpressing cells (Figure 3B), indicating that the N-cadherin-LRP5 interaction negatively controls ERK and PI3K signalling. N-cadherin silencing using si-RNA also increased ERK and PI3K phosphorylation in Flag cells compared to a non-relevant siRNA, indicating that endogenous N-cadherin negatively controls these pathways (Figure 3C). To determine the functional relevance of these findings, we tested the effects of ERK and PI3K inhibitors on cell proliferation induced by Wnt. As shown in Figure 3D, the positive effect of Wnt3a (15% CM) on cell growth was abolished by wortmannin (10 µM) and U0126 (10 µM) which are pharmacologic inhibitors of PI3K and MEK, respectively, in both control and N-cadherin overexpressing cells. These results indicate that ERK and PI3K signalling pathways are functionally involved in the altered cell growth induced by N-cadherin in osteoblasts.

**Figure 4:**
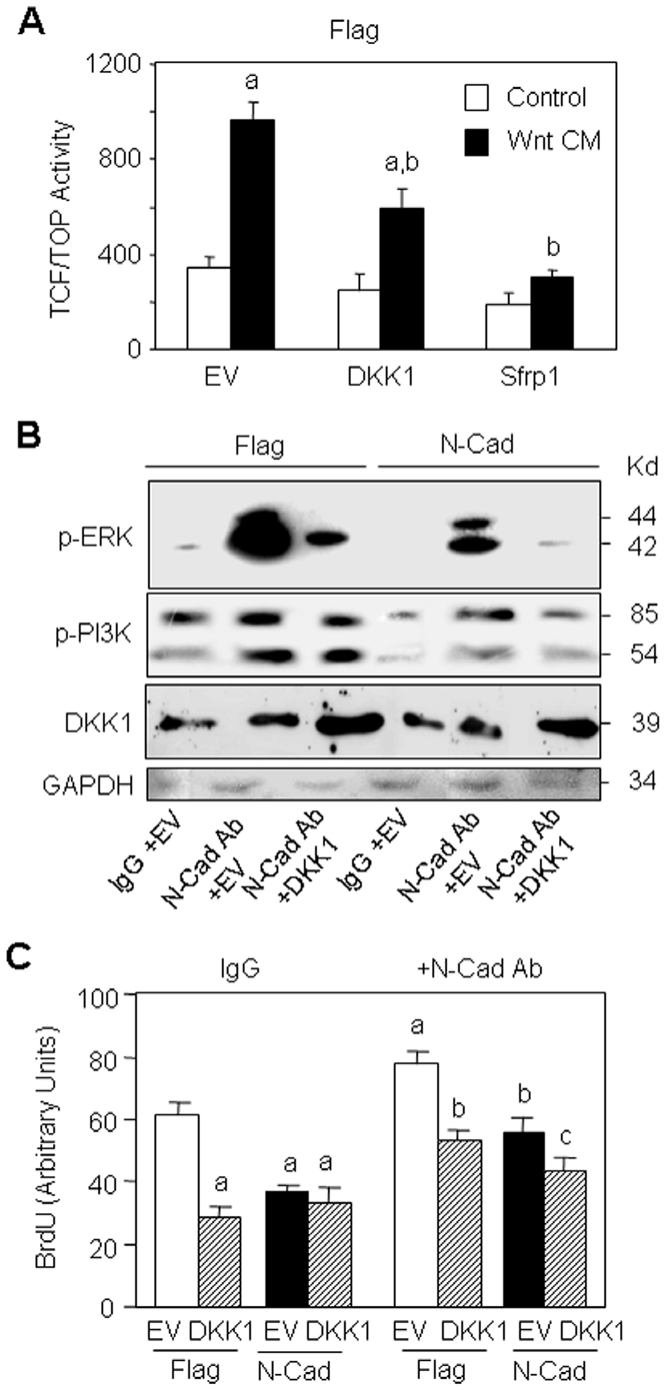
The Wnt inhibitor DKK1 abolishes ERK and PI3K signalling restored by N-cadherin blockade. (A) Control (Flag) cells were transiently transfected with empty vector (EV) or DKK1, or treated with the Wnt antagonist Sfrp1 in the presence or absence of Wnt3a CM and TCF/TOP transcriptional activity was determined. Means are +/− SD. Values that are significantly different are indicated (a, *P*<0.05 vs EV -Wnt treated cells; b, *P*<0.05 vs EV Wnt treated cells). (B) Flag and N-Cad cells transiently transfected with empty vector (EV) or DKK1 were treated with the bloking N-cadherin antibody (N-Cad Ab) or control antibody (IgG) for 24 hours and DKK1 levels and ERK and PI3K signalling were analysed by Western-blot. GAPDH was used as loading control. (C) Flag and N-Cad overexpressing cells transfected with empty vector (EV) or DKK1 were treated with the N-cadherin antibody for 24 hours to restore ERK and PI3K signalling or with control antibody (IgG), and cell replication was determined (a, *P*<0.05 vs EV Flag cells; b, *P*<0.05 vs N-Cad Ab treated EV Flag cells; c, *P*<0.05 vs N-Cad Ab treated EV N-Cad cells).

We then sought to determine the implication of canonical Wnt signalling in the altered ERK and PI3K signalling induced by N-cadherin-LRP5 interaction. To this goal, cells were treated with the neutralizing N-cadherin antibody to restore ERK and PI3K signalling and the cells were then transfected with the Wnt inhibitor DKK1. Transient transfection with DKK1 efficiently reduced β-catenin transcriptional activity, as determined by the TCF/TOP assay (Figure 4A). Treatment with Sfrp1 which binds and antagonizes Wnt proteins [7] also abolished the response to Wnt3a in these cells (Figure 4A). As shown in Figure 4B, the N-cadherin antibody increased ERK and PI3K signalling in control and N-cadherin cells, confirming our previous data. Transient transfection with DKK1 effectively increased DKK1 protein levels and abolished the restoration of ERK and PI3K activation induced by the N-cadherin antibody (Figure 4B). These results indicate that the altered ERK and PI3K signalling in N-cadherin overexpressing cells results in large part from attenuation of Wnt signalling. We then determined the functional role of Wnt signalling in the altered cell proliferation induced by N-cadherin. As shown in Figure 4C, transient transfection with DKK1 reduced cell proliferation in control cells. Neutralization of N-cadherin with the antibody increased cell growth and this effect was reduced by DKK1 transfection in both control and N-cadherin overexpressing cells (Figure 4C). The finding that DKK1 abrogates the effect of N-cadherin neutralizing antibody on ERK and PI3K signalling and cell growth confirm that Wnt signalling is implicated in the altered ERK and PI3K signalling induced by N-cadherin-LRP5 interaction in these cells.

**Figure 5:**
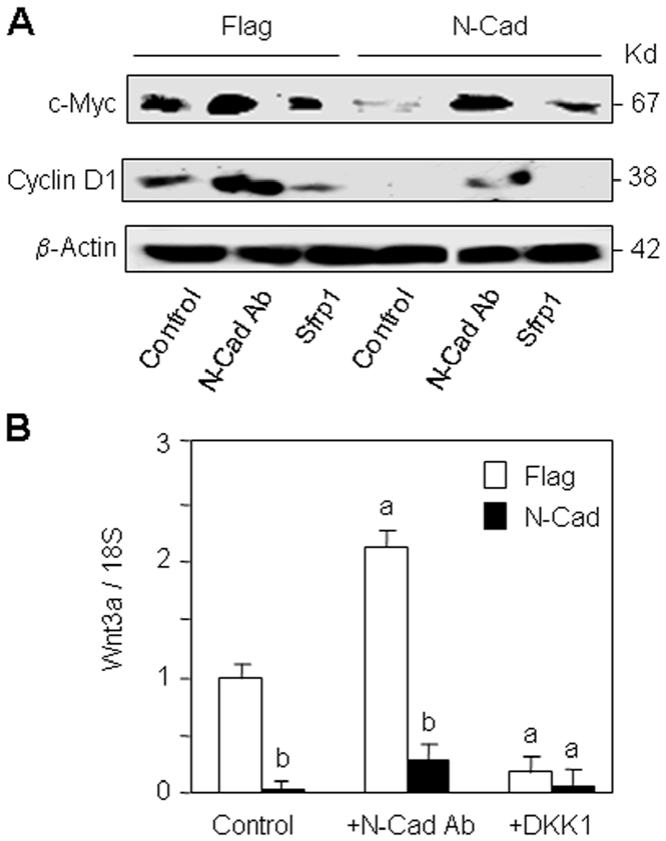
The altered cell proliferation induced by N-cadherin overexpression involves Wnt-responsive genes. (A) Control (Flag) and N-cadherin (N-Cad) overexpressing cells were treated with the blocking N-cadherin antibody, control antibody (IgG) or the Wnt antagonist Sfrp1 for 24 hours and the levels of the Wnt-responsive proteins c-Myc and cyclin D1 were analysed by Western-blot. β-actin was used as loading control. (B) Flag and N-Cad overexpressing cells were treated with N-cadherin antibody (N-Cad Ab) or IgG, or transiently transfected with the Wnt antagonist DKK1 and Wnt3a mRNA levels were determined by qPCR analysis at 24 hours. Means are +/− SD. Values that are significantly different are indicated (a, *P*<0.05 vs untreated Flag cells; b, *P*<0.05 vs corresponding Flag cells).

To further confirm the implication of Wnt signalling in the altered cell proliferation induced by N-cadherin-LRP5 interaction, we analysed the expression of Wnt-responsive genes in N-cadherin overexpressing cells. We first looked for changes in c-myc and cyclin D1 that are important target genes for the Wnt canonical pathway [43], [44]. As shown in Figure 5A, western blot analysis showed that both c-myc and cyclin D1 protein levels were markedly decreased in N-cadherin overexpressing cells compared to control (Flag) cells. We found that the N-cadherin antibody increased decreased c-myc and cyclin D1 levels whereas the Wnt antagonist Sfrp1 had opposite effects (Figure 5A). These results indicate that N-cadherin downregulates c-myc and cyclin D1 expression and further suggest the implication of Wnt signalling in this effect. Because Wnt3a is an important target gene for Wnt signalling, we investigated the effect of N-cadherin overexpression on Wnt3a ligand expression. We found that forced expression of N-cadherin nearly abolished endogenous Wnt3a mRNA expression compared to control cells (Figure 5B). Blockade of N-cadherin with the antibody increased Wnt3a expression in both control and N-cadherin overexpressing cells, indicating that N-cadherin-LRP5 interaction negatively controls endogenous Wnt3a expression in these cells. Transient transfection with DKK1 greatly reduced Wnt3a expression (Figure 5B), indicating that N-cadherin-LRP5 interaction negatively controls Wnt3a expression via alteration of canonical Wnt signalling. This indicates that in addition to negatively interact with LRP5, N-cadherin inhibits Wnt signaling by reducing endogenous Wnt ligand expression.

**Figure 6:**
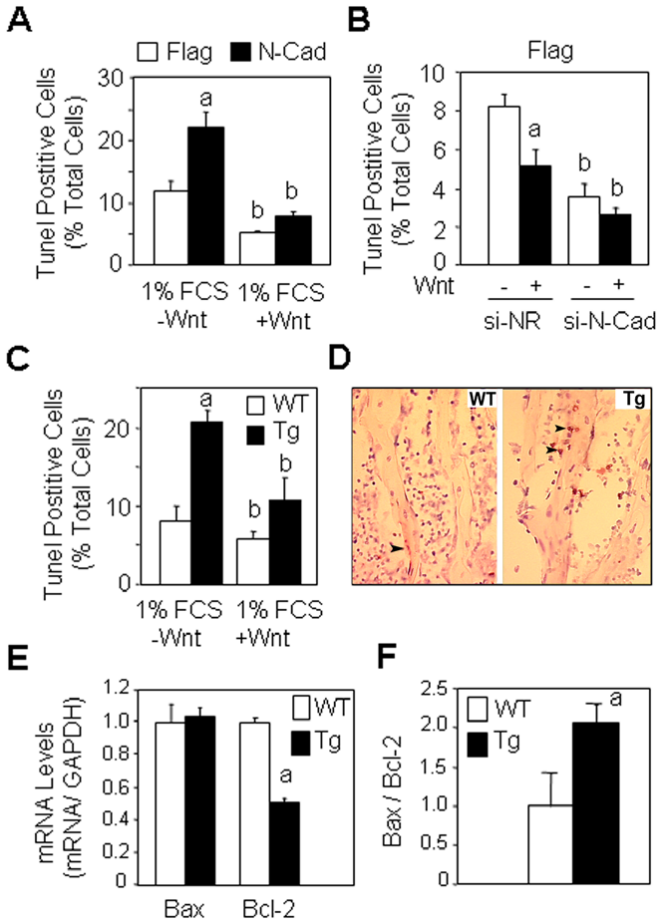
Forced expression of N-cadherin decreases cell survival. (A) Control (Flag) and N-cadherin (N-Cad) overexpressing cells cultured in serum deprived (1% FCS) medium were treated with Wnt3a (15% CM) for 24 hours and the number of TUNEL-positive cells was determined. Means are +/− SD (a, *P*<0.05 vs -Wnt Flag cells; b, *P*<0.05 vs corresponding Flag cells). (B) N-cadherin silencing decreases osteoblast apoptosis. Flag cells were transfected with N-cadherin si-RNA or a non relevant si-RNA (si-NR) and treated with Wnt3a CM (15%) for 24 hours in serum deprived (1% FCS) medium and cell replication was determined. (a, *P*<0.05 vs -Wnt si-NR cells; b, *P*<0.05 vs corresponding Flag cells). (C) Osteoblasts isolated from calvaria in wild type (WT) or N-cadherin transgenic mice (Tg) were cultured in serum deprived (1% FCS) medium and treated with Wnt3a (15% CM) for 24 hours and the number of TUNEL-positive cells was determined (a, *P*<0.05 vs WT cells; b, *P*<0.05 vs corresponding WT cells. (D) Histologial sections of tibias showing increased cell apoptosis in Tg mice compared to WT mice, as revealed by TUNEL staining (brown nuclei) in osteoblasts (Ob, arrows) (x250). (E, F) Decreased Bcl-2 mRNA levels and increased Bax/Bcl-2 ratio in tibias of Tg mice compared to WT mice (a, *P*<0.05 vs WT mice).

Having shown that N-cadherin negatively controls cell growth, we then sought to determine the role of N-cadherin on cell survival in osteoblasts. We first analysed the effect of N-cadherin overexpression on cell death induced by serum deprivation. As shown in Figure 6A, forced expression of N-cadherin increased the number of TUNEL-positive cells compared to control cells. This effect was partly dependent on canonical Wnt signalling since treatment with Wnt (15% CM) reduced cell apoptosis in both control and N-cadherin overexpressing cells (Figure 6A). To confirm the role of N-cadherin in the control of osteoblast survival, control (Flag) cells were treated with si-N-cadherin or a non-relevant si-RNA and cell survival was determined in serum deprived conditions. As shown in Figure 6B, N-cadherin silencing decreased the number of TUNEL-positive cells in the presence or absence of Wnt, further indicating that endogenous N-cadherin negatively controls cell survival in normal osteoblasts. To determine whether the negative effect of N-cadherin may be relevant to bone *in vivo*, cell death was analysed *ex vivo* in calvaria osteoblasts isolated from 1.5 month old wild-type and N-cadherin transgenic mice cultured in serum deprived conditions. As shown in Figure 6C, N-cadherin transgenic cells displayed increased cell apoptosis compared to wild-type cells in the presence or absence of Wnt, indicating that N-cadherin overexpression induces a cell autonomous defect in cell survival. To confirm the relevance of these findings *in vivo*, we performed a histological analysis of cell apoptosis in bones from N-cadherin transgenic and wild type mice. Histological analysis revelated a higher number of TUNEL-positive osteoblasts (brown nuclei) in tibias of transgenic N-cadherin mice compared to wild type mice (Figure 6D, arrows). Accordingly, we found that mRNA expression level of the anti-apoptotic protein Bcl-2 was decreased by 50% in tibias of transgenic compared to wild type mice whereas expression of the pro-apoptotic protein Bax was unchanged (Figure 6E). Consequently, the Bax/Bcl-2 ratio was higher in tibias of transgenic mice compared to wild type mice, reflecting increased apoptosis (Figure 6F). These results demonstrate that the negative effect of N-cadherin on osteoblast survival *in vitro* is relevant to bone *in vivo*.

**Figure 7:**
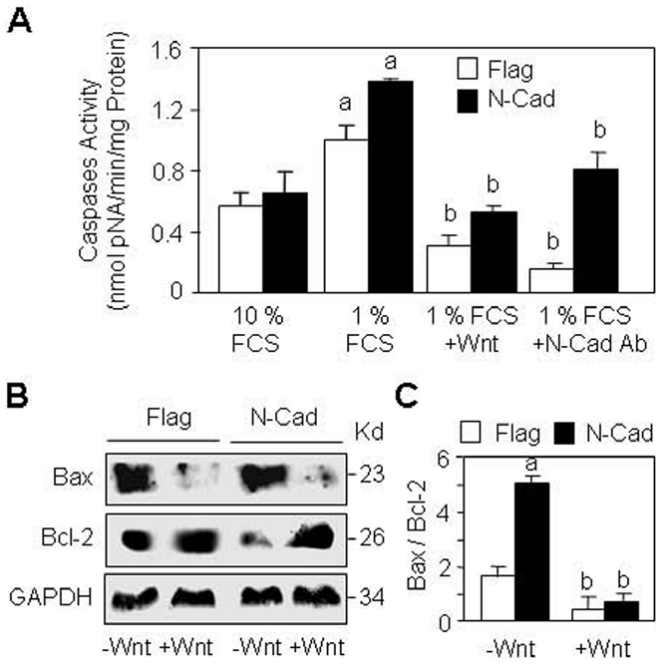
Mechanisms by which N-cadherin decreases cell survival. (A) Control (Flag) and N-Cadherin overexpressing cells (N-Cad) were cultured in survival conditions (10% FCS) or serum deprived (1% FCS) medium, treated with Wnt3a (15% CM), blocking N-cadherin antibody or control antibody (IgG) for 24 hours and effector caspase activity was determined. Means are +/− SD (a, *P*<0.05 vs corresponding 10% FCS; b, *P*<0.05 vs corresponding untreated cells. (B) Flag and N-Cad cells were cultured in serum deprived (1% FCS) medium, treated with Wnt3a (15% CM) for 24 hours, and the levels of Bax and Bcl-2 proteins were analysed by Western blot using GAPDH as loading control. (C) Quantification of western blots showing the increased Bax/Bcl-2 ratio in N-Cad cells which was abolished by Wnt3a (a, *P*<0.05 vs untreated Flag cells; b, *P*<0.05 vs corresponding untreated cells).

We then investigated the underlying mechanisms involved in the increased cell apoptosis induced by N-cadherin. We found that N-cadherin overexpression increased effector caspases 3, 6, 7 activity, and this effect was abrogated by Wnt (15% CM) and N-cadherin blockade (Figure 7A), indicating that the increased cell apoptosis induced by N-cadherin overexpression is caspase-dependent and related to N-cadherin-LRP5 interaction. To further determine the implication of Wnt signalling, cells were treated with Wnt3a (15% CM) and the protein levels of Bax and Bcl-2 were determined. As shown in Figure 7B, N-cadherin overexpressing cells showed reduced Bcl-2 levels compared to control cells, and treatment with canonical Wnt3a restored Bcl-2 levels in these cells. Quantification of western blots confirmed that the increased Bax/Bcl-2 ratio induced by N-cadherin overexpression was normalized by Wnt3a (Figure 7C). Altogether, these results indicate that N-cadherin-LRP5 interaction decreases cell survival in osteoblasts and that this effect is dependent on alteration of Wnt signalling.

**Figure 8:**
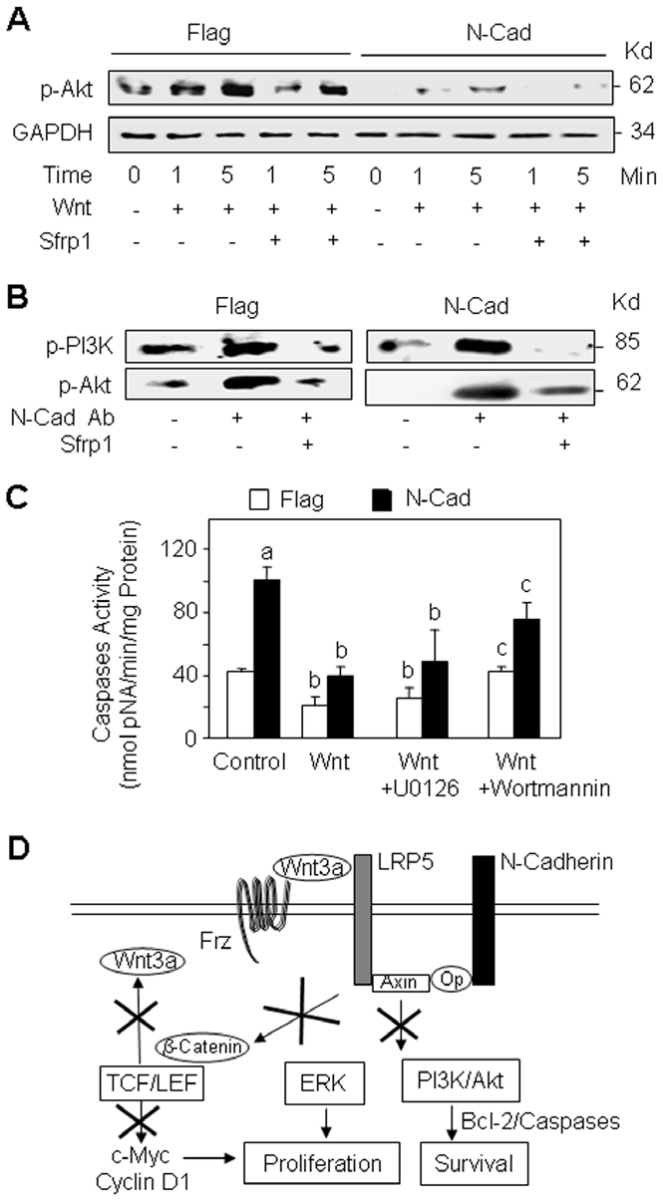
The altered cell survival induced by N-cadherin overexpression is Wnt- and PI3K/Akt-dependent. (A) Control (Flag) and N-cadherin (N-Cad) overexpressing cells cultured in serum deprived (1% FCS) medium were treated with canonical Wnt3a (15% CM) or the Wnt antagonist Sfrp1 for 1 to 5 minutes and Akt signalling was analysed by Western-blot. GAPDH was used as loading control. (B) Flag and N-Cad overexpressing cells cultured in serum deprived (1% FCS) medium were treated with the blocking N-cadherin antibody, control antibody (IgG) or Sfrp1 and PI3K/Akt signalling was analysed by Western-blot. (C) Flag and N-Cad cells cultured in serum deprived (1% FCS) medium were treated with canonical Wnt3a (15% CM) for 24 hours in the presence or absence of the MEK inhibitor U0126 or the PI3k inhibitor wortmannin, and effector caspase activity was determined. Means are +/− SD. Values that are significantly different are indicated (a, *P*<0.05 vs untreated Flag cells; b, *P*<0.05 vs corresponding untreated cells; c, *P*<0.05 vs corresponding Wnt3a-treated cells). (D) Proposed mechanisms by which N-cadherin acts as a negative regulator of cell proliferation and survival in osteoblasts. N-cadherin interaction with LRP5 (and other proteins: OP) decreases the expression of the autocrine/paracrine Wnt3a ligand and Wnt responsive genes c-Myc and cyclin D1, and causes attenuation of Wnt, ERK and PI3K/Akt signalling, resulting in inhibition of cell proliferation and survival.

Because Akt signalling is an important pathway controlling cell survival [45] and cross talks between Wnt and Akt signalling have been reported in osteoblasts [9], [24], [46], we determined the implication of Akt in the altered cell survival induced by N-cadherin-LRP5 interaction in osteoblasts. As shown in Figure 8A, we found that p-Akt levels were markedly decreased in N-cadherin overexpressing cells cultured in serum deprived medium compared to control cells. Treatment with Wnt3a (15% CM) greatly increased p-Akt levels in control (Flag) cells and to a much lower extent in N-cadherin overexpressing cells (Figure 8A). The effect of the Wnt conditioned medium at this time point was not due to other components present in the CM since the Wnt inhibitor Sfrp1 abolished the effect of CM (Figure 8A). These results indicate that N-cadherin overexpression markedly affects Wnt3a-dependent Akt phosphorylation in these cells. To analyse the implication of N-cadherin-LRP5 interaction in this effect, cells were treated with the neutralizing N-cadherin antibody and PI3K/Akt signalling was determined by Western blot analysis. N-cadherin blockade greatly increased p-PI3K and p-Akt levels in both control and N-cadherin overexpressing cells and this effect was abrogated by the addition of the Wnt antagonist Sfrp1 (Figure 8B). These results further indicate that the altered PI3K/Akt signalling induced by N-cadherin overexpression is dependent on Wnt signalling. To establish the functional role of the altered PI3K/Akt and Wnt signalling in the altered cell survival induced by N-cadherin overexpression, cells were treated with Wnt3a (15% CM) and the MEK or PI3K inhibitors and effector caspase activity was determined. As shown in Figure 8C, treatment with Wnt3a (15% CM) decreased effector caspase activity in both control and N-cadherin overexpressing cells. The PI3K inhibitor, but not the MEK inhibitor, blunted the effect of Wnt3a on effector caspase activity (Figure 8C). These results indicate that Wnt and PI3K/Akt signalling pathways are involved in the altered cell survival induced by N-cadherin. Overall, our results indicate that N-cadherin controls cell proliferation and survival in osteoblasts by mechanisms involving alteration of autocrine/paracrine Wnt3a ligand expression and attenuation of Wnt, ERK and PI3K/Akt signalling pathways (Figure 8D).

## Discussion

It is well documented that Wnt signalling plays an important role in the control of cell and tissue development. Consistent with this essential function, Wnt signalling is tightly controlled by several intracellular and secreted antagonists [6], [7], [47]. We recently showed that N-cadherin acts as a new antagonist of Wnt signalling by acting as a partner of LRP5/6 Wnt co-receptors in osteoblasts [39]. In this study, we demonstrate a novel role for N-cadherin in the control of cell proliferation and survival. We show that forced expression of N-cadherin downregulates cell proliferation and increases cell apoptosis in osteoblasts *in vitro* and *in vivo*, and that these negative effects of N-cadherin are related to N-cadherin-LRP5 interaction. These results support the idea that N-cadherin negatively controls cell growth and survival in addition to inhibit cell differentiation and function in osteoblasts.

One major question is how N-cadherin interaction may negatively regulate cell proliferation and survival. We found that the decreased cell proliferation and survival induced by N-cadherin overexpression was abrogated by N-cadherin blockade, which reverses N-cadherin-LRP5 interaction in osteoblasts [39] and was partly restored by Wnt3a, indicating that N-cadherin-LRP5 mediated alteration of canonical Wnt signalling contributes to the negative effect of N-cadherin on cell growth. Consistent with this idea, we showed that N-cadherin overexpression reduced the expression of Wnt-responsive genes such as c-myc and cyclin D1 which control cell growth. More importantly, N-cadherin overexpression markedly reduced the expression of canonical Wnt3a, an effect that was partly reversed by N-cadherin blockade. This strongly suggests that N-cadherin-LRP5 interaction downregulates cell proliferation in part by reducing endogenous Wnt3a expression and subsequent canonical Wnt signalling. This provides a molecular mechanism whereby N-cadherin ultimately controls osteoblast proliferation via alteration of a Wnt3a autocrine/paracrine loop (Figure 8D). The negative role of N-cadherin on Wnt3a expression may have important functional implications in the control of bone formation since the Wnt3a autocrine/paracrine loop is an essential mechanism involved in the action of physiological anabolic factors [22].

Wnt signalling is known to affect cell growth and survival in several systems in part by regulating ERK and PI3K signalling [9], [10]. Notably, the PI3K/Akt signalling cascade plays a key role in the control of cell proliferation and survival [48]. We and others previously showed that PI3K/Akt is an important signalling pathway involved in the control of osteoblast survival [24], [46], [49], [50]. Previous studies revealed that engagement of E-cadherin in homophylic calcium-dependent cell-cell interactions results in rapid PI3K-dependent activation of Akt, indicating that E-cadherin can initiate outside-in signal transducing pathways that regulate the activity of PI3K and Akt [51]. In contrast, we show here that N-cadherin overexpression downregulates PI3K and Akt activity which mediates in part the negative effect of N-cadherin on osteoblast growth and survival. Several arguments support the idea that these alterations of PI3K/Akt signalling are in part dependent on Wnt signalling. First, the negative effect of N-cadherin overexpression on ERK and PI3K pathways and cell growth was restored by N-cadherin blockade which reverses N-cadherin-LRP5 interaction. Second, inhibition of Wnt signaling using DKK1 or Sfrp1 abolished the ability of N-cadherin blockade to restore ERK and PI3K phosphorylation and cell proliferation in N-cadherin overexpressing cells. Third, cell replication induced by Wnt3a was inhibited by pharmacological inhibition of ERK and PI3K, indicating that these kinases act downstream of Wnt3a to promote osteoblastic cell growth. Finally, we found that the decreased cell survival induced by N-cadherin overexpression was reversed by Wnt and antagonized by PI3K inhibition. These observations support our hypothesis that alterations of ERK and PI3K signalling are secondary to inhibition of Wnt signalling induced by N-cadherin, resulting in the observed alterations of cell growth and survival.

In summary, the present study reveals a novel role for N-cadherin in the control of osteoblastogenesis *in vitro* and *in vivo*. Our data indicate that N-cadherin controls osteoblast proliferation and survival via attenuation of autocrine Wnt3a ligand expression and alteration of at least three signalling pathways in osteoblasts (Figure 8D).

## Materials and Methods

### Cell cultures, transfections and reagents

MC3T3-E1 cells (ATCC) were stably transfected with N-cadherin Flag-tagged cloned in PCDNA 3.1 and selected using G418 (Calbiochem, San Diego, USA) as previously described [39] and over-expression was verified by Western blot analysis. Transient transfection with DKK1 (Galapagos, Romainville, France) and TCF/TOP transcriptional activity were performed as described previously [39], [52]. Tibias from 1.5 month old female N-cadherin transgenic and wild type mice and primary osteoblasts isolated from calvarias by sequential collagenase digestion were obtained as described [39]. Wnt3a-conditioned medium (CM) and si-RNAs were prepared as described [39], [52]. Recombinant human Sfrp1 was from R&D, Minneapolis, MN, USA, and blocking N-cadherin antibody and pharmacologic inhibitors of PI3K (wortmannin) and MEK (U0126) were from Sigma (USA).

### Cell proliferation and apoptosis

For analysis of cell replication, cells were plated at 2000 cells/dish in 96 wells, treated as indicated and cell replication was determined using the BrdU ELISA assay (Roche, France) and cell number. In some experiments, cells were cultured in serum deprived (1% FCS) medium to induce apoptosis and treated with wortmannin (10 µM), U0126 (10 µM), the blocking N-cadherin antibody (10 µg/ml) or Wnt3a conditioned medium (15%) for 24 hours and caspases-3, -6, -7 activity was determined as described [53]. DNA degradation was analysed by TUNEL analysis using the Apop Tag Kit (Chemicon USA) according to manufacturer's recommendations. The number of TUNEL-positive cells was expressed as %of total cells.

### Western blot, immunoprecipitation and immunohistochemical analyses

For Western blot analysis, 30 µg of proteins were loaded on Ge-Ba gel (4–12%) (Gene Bio Application Ltd, Kfar Hanagid, Israel). After electrophoresis, transferred proteins were revealed with anti-Flag (Sigma-Aldrich), anti-c-Myc (AbCam, Cambridge, UK), anti-N-cadherin or anti-LRP5 (Cell Signalling, Denver, USA), detected using a secondary horseradish peroxidase antibody (Beckman Coulter, Fullerton, USA) and quantified using Quantity One software (BioRad). Immunoprecipitation analysis was performed using microMACS protein A/G microbeads magnetic separation (Miltenyi Biotech Auburn CA, USA) according to manufacturer's recommendations. Briefly 100 µg of total protein were incubated 30 minutes on ice with 2 µg of the indicated antibody or immunoglobulin fraction negative control (Dako, Glostrup, Denmark) and 20 µl of protein A/G magnetically labelled. The magnetically labelled immune complex was passed over a micro-column placed in a magnetic field. The complex bound was washed with lysis buffer, and the immunoprecipited protein was eluted from the column with SDS gel loading buffer ready for western blot assay. Immunohistochemistry was performed on decalcified serial sections of tibiae from 1.5 month female N-cadherin and wild type mice using the PK-6101 stain kit (Vector, Abcys, France) and primary polyclonal antibodies for KI67 (SantaCruz, USA) and TUNEL labelling (Chemicon) used at 1∶100 dilution, according to the manufacturer's instructions. Sections were then counterstained with toluidine blue and the same metaphyseal area was analysed (magnification×250).

### Quantitative real-time PCR analysis

For RNA preparation from tibias obtained from 1.5 month old N-cadherin transgenic and wild type mice, the bone marrow was flushed out and total RNA was isolated using Trizol (InVitrogen) and cleaned using an RNAeasy minikit (Quiagen, Courtaboeuf, France). Quantitative real-time PCR analysis of total RNA from tibias and cultured cells was performed using Roche Light Cycler and Absolute SYBR Green capillary mix (Abgene, Epson, UK). The sets of primers were for Wnt3a: forward 5′-CTTAGTGCTCTGCAGCCTGA-3′, reverse 5′-AGTGCTCAGAGAGGAGTACT-3′; for Bax: forward 5′-CTG AGC GGC TGC TTG TCT-3′, reverse5′-GGT CCC GAA GTA GGA GAG GA-3′ ; for Bcl-2: forward 5′-GTA CCT GAA CCG GCA TCT G-3′, reverse 5′-GGG GCC ATA TAG TTC CAC AA-3′ and for 18S: forward 5′-CGGCTACCACATCCAAGGAA-3′; reverse 5′-GCTGGAATTACCGCGGCT-3′.

### Statistical analysis

The experiments were repeated 3 times with at least 6 replicates per experiment. Data are expressed as mean +/− SD and analyzed using the statistical package super-ANOVA (Macintosh, Abacus concepts, Inc., Berkeley, CA).
